# Protective Effect of Carvedilol Against Oxidative Stress Induced by Palmitic Acid in Primary Rat Hepatocytes

**DOI:** 10.1002/cbf.70057

**Published:** 2025-02-09

**Authors:** Sandra A. Serna Salas, Turtushikh Damba, Manon Buist‐Homan, Han Moshage

**Affiliations:** ^1^ Department of Gastroenterology and Hepatology, University Medical Center Groningen University of Groningen Groningen the Netherlands; ^2^ School of Pharmacy Mongolian National University of Medical Sciences Ulaanbaatar Mongolia; ^3^ Department of Laboratory Medicine, University Medical Center Groningen University of Groningen Groningen the Netherlands

**Keywords:** antioxidants, carvedilol, ER stress, fatty acids, hepatocytes, lipotoxicity, MASLD, oxidative stress

## Abstract

Hepatocyte lipotoxicity (HL) is an important factor in the pathogenesis of Metabolic Dysfunction‐Associated Steatotic Liver Disease (MASLD). It is defined as the detrimental effects of exposure to (excessive) amounts of toxic lipid species, leading to increased mitochondrial β‐oxidation, oxidative stress (OxS), and organellar dysfunction. Carvedilol (CV) is a β‐adrenergic blocker with antioxidant properties. To elucidate whether CV protects hepatocytes against lipotoxicity induced by palmitic acid (PA) by reducing OxS and endoplasmic reticulum (ER) stress. Primary rat hepatocytes (rHep) were used. Lipotoxicity was induced by PA (1 mmol/L). Cell damage was evaluated by Sytox Green staining. Mitochondrial generation of reactive oxygen species (mROS) was assessed by MitoSox. mRNA and protein expression were measured by qPCR and Western blot, respectively. Lipid accumulation was measured by Oil Red O staining and triglyceride (TG) content. PA induced cell death in > 80% of cells and increased mROS generation. PA increased mRNA expression of ER stress markers CHOP and sXBP1 and slightly increased lipid accumulation. Expression of the β‐oxidation‐related gene Cpt1a was increased. CV (10 µmol/L) significantly reduced PA‐induced cell death to control levels (< 8% of total cells), and mROS generation and expression of the mitochondrial antioxidant enzymes Sod2 and Cat were increased by 40% by CV in the presence of PA. CV did not change the expression of ER stress markers. CV, added before PA, protects rHep against PA‐induced cytotoxicity by reducing OxS and increasing the expression of antioxidant enzymes without any additional protective effect on ER stress or lipid accumulation.

## Summary

This is the first study demonstrating that CV protects against palmitate‐induced cell death in hepatocytes via scavenging reactive oxygen species without modulating lipid accumulation or reducing endoplasmic reticulum stress.

## 1 Introduction

Hepatocyte lipotoxicity (HL) is an important factor in the onset and progression of Metabolic Dysfunction‐Associated Steatotic Liver Disease (MASLD) [1]. Steatosis in MASLD is defined as the presence of lipids in more than 5% of the hepatocytes, and lipotoxicity is defined as the damage and/or cell death that occurs as the result of exposure to toxic lipid species and the presence of intra‐hepatocytic lipids during the development of MASLD [2, 3]. Hepatocytes are the primary target of lipotoxic lipid species, but other cells, both intrahepatic and extrahepatic, are also responsive to the effects of these lipids. MASLD can progress to more advanced stages characterized by inflammation such as NASH (nonalcoholic steatohepatitis) and eventually to fibrosis and cirrhosis [4, 5, 6, 7, 8]. The incidence of MASLD worldwide is high but varies depending on the geographical area. In the United States, the prevalence is 34%, whereas in Europe it is around 25%. Lipotoxicity occurs when lipid homeostasis is disturbed [9]. In MASLD, excessive levels of free fatty acids (FFAs) due to increased dietary intake, *de novo* lipogenesis, and efflux from peripheral (adipose) tissue lead to increased exposure and uptake of FFAs in hepatocytes. This, in turn, leads to increased mitochondrial β‐oxidation, generation of reactive oxygen species (ROS), and organellar dysfunction, in particular dysfunction of mitochondria and endoplasmic reticulum (ER) [10]; Geng Y et al. 2020 [11]. In addition to the detrimental effects on mitochondrial metabolism, lipid toxicity is also related to lipid peroxidation. Lipid peroxidation generates lipid hydroperoxides triggering deleterious effects for the cell such as damage to the cell membrane, inactivation of membrane‐bound receptors and enzymes, and impairment of tissue permeability [12, 13, 14]. Moreover, lipid peroxides can generate additional ROS [12]. When the generation of ROS exceeds the antioxidant capacity of cells, oxidative stress (OxS) occurs. Some products generated by ROS, such as 4‐hydroxy‐*trans*‐2‐nonenal (HNE), can interact with chaperones from the ER, which assist in protein folding and thus induce ER stress, resulting in inflammation and eventually cell death [15]. Drug‐based therapy does not exist for MASLD and its advanced stages (NASH). The only life‐saving treatment for end‐stage liver disease as a result of MASLD is liver transplantation. Therefore, it is vital to elucidate the pathogenic mechanisms of MASLD to identify novel targets for (therapeutic) intervention. Since OxS is a hallmark of lipotoxicity, the identification of compounds that can alleviate these detrimental effects is urgently needed. Carvedilol (CV) is a compound used to treat hypertension and congestive heart failure. It is a combined β‐ and α‐adrenergic blocker, presenting 10‐fold more affinity for β‐adrenergic receptors (β‐AR). CV also appears to be a good candidate to treat portal hypertension in patients with cirrhosis [16, 17]. Furthermore, CV and its secondary metabolites have potent antioxidant activity [18, 19]. In animal models of chronic liver diseases, CV demonstrated anti‐fibrotic and anti‐inflammatory properties, attributed to its antioxidant activity [20, 21]. Less is known about CV as a therapeutic agent for MASLD and/or NASH. However, since CV is an antioxidant and lipotoxicity is linked to high production of ROS, CV could be a potential agent in the treatment of MASLD and NASH. Moreover, CV inhibits the cellular response of β‐ARs, and these receptors are known to participate in lipid metabolism via enhancing fatty acid catabolism by lipid peroxidation [22, 23, 24, 25]. In this study, the following hypothesis was tested: CV protects hepatocytes against palmitate‐induced lipotoxicity by attenuating ROS generation and/or lipid accumulation in hepatocytes.

## 2 Materials and Methods

### 2.1 Animals

The research described in this study was prospectively reviewed and approved by the Institutional Animal Care and Use of Laboratory Animals Committee of the University of Groningen (DEC‐RUG), protocol AVD10500202115139, following the Dutch law on the welfare of laboratory animals. All animal experiments were performed according to the Dutch law concerning the welfare of laboratory animals, and the guidelines of Institutional Animal Care and Use of Laboratory Animals Committee of the University of Groningen (DEC‐RUG) were followed.

### 2.2 Hepatocyte Isolation and Culture

Specified pathogen‐free male Wistar rats (150–200 g; 6–8 weeks) were purchased from Charles River Laboratories Inc. (Wilmington, Massachusetts, the United States) and kept for acclimatization for at least 1 week. Animals were housed in polypropylene cages at room temperature (25 ± 2°C) with standard bedding, regular (12‐h light/12‐h dark) day/night cycle, and free access to standard laboratory chow and water at the animal facility center of the University Medical Center of Groningen. During isolation, anesthesia was first induced through the use of an isofluorane (5%) chamber for 5 min. For anesthesia, a combination of ketamine (100 mg/mL, 60 mg/kg body weight) and medetomidine hydrochloride (1 mg/mL, 0.5 mg/kg body weight) was used. All efforts were made to minimize animal suffering and to reduce the number of animals used.

Primary rat hepatocytes (rHep) were isolated from male Wistar rats (250–300 g; 9–11 weeks) following the two‐step collagenase liver perfusion method exactly as described previously [26]. Briefly, the rat liver was perfused in situ via the portal vein with calcium‐ and magnesium‐free Hanks' balanced salt solution, followed by perfusion with collagenase to dissociate the liver as described previously. Hepatocytes were isolated and washed by repeated (3×) differential centrifugation (50 × g, 5 min) of the resulting cell suspension. The hepatocytes were seeded on various tissue culture plastics and coverslips (CS) at a density of 125.000/cm^2^. Trypan blue was used to determine cell viability, and only hepatocytes with a viability of 80% or higher were used for experiments. Purity was checked using morphological criteria (hepatocytes acquire a characteristic cuboidal/hexagonal morphology after attachment, and purity was always greater than 97%). Rat hepatocytes were seeded in William's E medium (Invitrogen, Breda, the Netherlands) containing 5% heat‐inactivated fetal calf serum (Thermo Fisher Scientific), 100 U/mL penicillin (Lonza, Vervier, Belgium), 10 μg/mL streptomycin (Lonza), 250 ng/mL fungizone (Lonza), and 50 μg/mL of gentamicin (Invitrogen) and cultured in a humidified atmosphere containing 5% CO_2_ at 37°C. Experiments started 4 h after seeding.

### 2.3 Experimental Design

To induce lipotoxicity, rHep were seeded in 6‐ and 12‐well plates and treated with palmitic acid (PA; 1 mmol/L, Sigma‐Aldrich) dissolved in 10% FFA‐free bovine serum albumin (FFA‐free BSA, Sigma‐Aldrich) in phosphate‐buffered saline (PBS, Invitrogen). As a positive control for lipid accumulation, rHep were treated with a mixture of oleate and palmitate (final concentrations 500 μmol/L and 250 μmol/L, respectively) as described previously [27]. CV was used at a concentration of 10 μmol/L and added 30 min before palmitate and was tested as a preventive compound. Nontreated controls were treated with FFA‐free BSA alone.

### 2.4 Sytox Green Nuclear Staining

Sytox Green Nucleic acid Stain (Invitrogen, S7020) was used to determine necrotic cell death. Sytox Green only enters cells with leaky plasma membranes and accumulates in nuclei, indicative of (necrotic) cell death. After 24‐h treatment with PA and/or CV, rHep were incubated with Sytox Green Solution (1:40,000 in PBS) for 15 min in an incubator containing 5% CO_2_ at 37°C. Cell necrosis was visualized using a Leica fluorescence microscope with an excitation wavelength of 450–490 nm. H_2_O_2_ (1 mmol/L) was used as a positive control for necrosis. Images were analyzed by Image J software version 2.0.0‐rc‐69/1.52n.

### 2.5 Caspase 3/7 Activity Assay

Apoptotic cell death was measured by caspase 3/7 enzyme activity assay. After treatments, cells were washed twice with ice‐cold PBS (Life Technologies), followed by the addition of caspase cell lysis buffer. Caspase 3/7 enzyme activity was determined following the manufacturer's instructions, as described previously [28].

### 2.6 mRNA Isolation and Quantitative Polymerase Chain Reaction

mRNA was isolated from rHep using TRIzol reagent according to the manufacturer's protocol (Thermo Fisher Scientific). mRNA concentration was measured with a Nanodrop 2000c UV–VIS spectrophotometer (Thermo Fisher Scientific) and stored at −80°C until use. Reverse transcription was performed with 2.5 μg of total RNA using random nonamers and M‐MLV reverse transcriptase (Invitrogen, the United States). Subsequently, real‐time quantitative PCR was performed using the qPCR core kit master mix (Eurogentec, the Netherlands) on a 7900 H T Fast Real‐Time PCR System (Applied Biosystems, Foster City, California, the United States). Relative expression levels were normalized to 18S, used as an internal housekeeping gene, and presented as relative levels compared to the control condition. The control condition was set to 1. Sequences of PCR primers are listed in Table 1.

**Table 1:** Sequences of primers and probes used for real‐time PCR analysis.

Gene	Sense 5′3′	Antisense 5′3′	Probe 5′3′
*Cd36*	GATCGGAACTGTGGGCTCAT	GGTTCCTTCTTCAAGGACAACTTC	AGAATGCCTCCAAACACAGCCAGGAC
*Srebp‐1c*	GGA GCC ATG GAT TGC ACA TT	CCT GTC TCA CCC CCA GCA TA	CAG CTC ATC AAC AAC CAA GAC AGT GAC TTC C
*Dgat2*	GGGTCCAGAAGAAGTTCCAGAAG	CCCAGGTGTCAGAGGAGAAGAG	CCCCTGCATCTTCCATGGCCG
*PPARα*	CAC CCT CTC TCC AGC TTC CA	GCC TTG TCC CCA CAT ATT CG	TCC CCA CCA GTA CAG ATG AGT CCC CTG
*Cpt1a*	CAG TGG GAG CGA CTC TTC AAT	GCC CTC TGT GGT ACA CAA CAA	CCT GGG GAA GAG ACA GAC ACC ATC CAA C
*Atf4*	CTATCTCCATTCTACTACTACCAGATCGA	CCTGGGCCTCAGCTTCTCAT	CCCTGGAAGACCCACATCTGG CAG
*Chop*	TCCTGTCCTCAGATGAAATTGG	TCAAGAGTAGTGAAGGTTTTTGATTCT	CACCTATATCTCATCCCCA
*Grp78*	AAAGAAGGTCACCCATGCAGTT	CAATAGTGCCAGCATCCTTGT	ACTTCAATGATGCACAGCGGCAAGC
*Sxbp1*	GCT GAG TCC GCA GCA GGT	CCC AAA AGG ATA TCA GAC TCA GAA TC	CCC AGT TGT CAC CTC CCC AGA ACA TCT
18s	*Srebp‐1c*CGGCTACCACATCCAAGGA	CCAATTACAGGGCCTCGAAA	CGCGCAAATTACCCACTCCCGA

### 2.7 Protein Isolation, Quantification, and Western blot Analysis

Protein samples were prepared for Western blot analysis as described previously [29]. Protein concentrations were quantified using the Bio‐Rad protein assay (Bio‐Rad, Hercules, California, the United States) with BSA as a standard. 10–20 µg protein was used to perform a Western blot. Details of primary antibodies and dilutions are listed in Table 2. Appropriate horseradish peroxidase (HRP)‐conjugated secondary antibodies (dilution 1:2000; DAKO) were used for detection. Proteins were detected using Pierce ECL Western blot kit (Thermo Fisher Scientific), and images were captured using Chemidoc MR (Bio‐Rad) system. The intensity of bands was quantified with ImageJ version 2.0.0‐rc‐69/1.52n.

**Table 2:** Primary antibodies and dilutions.

Protein	Species	Dilution	Company
p‐JNK	Polyclonal rabbit	1:1000	Cell signaling
JNK	Polyclonal rabbit	1:1000	Cell signaling
eIF2α	Polyclonal rabbit	1:1000	Cell signaling
p‐eIF2α	Polyclonal rabbit	1:1000	Cell signaling

### 2.8 Immunofluorescence Microscopy

rHep were seeded on CS and treated as described in Section 2.3. After treatment, cells were fixed with 4% paraformaldehyde (Merck Millipore) and permeabilized with 1% Triton X‐100 followed by blocking with 2% BSA in PBS. Cells were incubated with primary antibody against SOD2 (Enzo Life Sciences, Brussels, Belgium) and labeled with secondary antibody Alexa Fluor 488 (Invitrogen). CS were mounted with Vectashield Antifade Mounting Medium with DAPI (Vector Laboratories, Gdynia, Poland). CS were air‐dried, sealed using nail polish, and stored at 4°C, protected from light until further use. Images were taken with a Leica Fluorescence microscope (Leica Microsystems, Wetzlar, Germany) and processed using ImageJ software version 2.0.0‐rc‐69/1.52n.

### 2.9 Measurement of ROS

rHep were seeded on 96‐well and 6‐well plates to measure and detect mitochondrial superoxide production. After attachment (4 h), cells were treated as described in Section 2.3 for 4 h. After treatment, cells were incubated with MitoSox‐Red (2.5 µmol/L) for 15 min at 37°C, protected from light. Next, cells were washed twice with warm PBS. Fluorescence was measured at Ex/Em wavelengths of 518/605 nm using a Bio‐Tek FL600 microplate fluorescence reader (Bio‐Tek). Images were taken with a Leica Fluorescence microscope (Leica Microsystems, Wetzlar, Germany). The MitoSox assay is a fluorescent probe that measures mitochondrial superoxide anion production and is not sensitive to other ROS or RNS species.

### 2.10 Analysis of Mitochondrial Membrane Potential

Mitochondrial membrane potential was assessed using JC‐10 staining following the manufacturer's protocol. In healthy cells, JC‐10 diffuses within the mitochondrial matrix and forms red fluorescent aggregates. When cells are damaged, the membrane potential decreases and JC‐10 diffuses out of the mitochondria as green fluorescent monomers. The ratio of red (aggregates)/green (monomers) fluorescence is a measure of mitochondrial membrane polarization. rHep were seeded in 6‐well plates and treated for 6 h with PA and CV as described before. Cells were incubated with JC‐10 at 100 µmol/L for 30 min, and aggregates and monomers were visualized using a Leica Fluorescence microscope (Leica Microsystems, Wetzlar, Germany). The mitochondrial membrane potential was shown as a ratio of JC‐10 aggregates over monomers.

### 2.11 Oil Red Staining and TG Measurement

rHep were cultured on CS and treated as described above. Next, cells were washed with PBS and fixated with 4% paraformaldehyde for 10–15 min. Oil Red O was used to stain the lipid droplets (Sigma‐Aldrich). Oil Red O solution (60% dissolved in isopropanol) was filtered and added to the fixated cells for 15 min. Hematoxylin was used to stain the nucleus. Cells were scanned by slide scanner Nanozoomer (Hamamatsu Photonics K.K., Shizuoka, Japan). Triglyceride (TG) content was quantified in lysed cells using a TG quantification kit (Abcam, Cambridge, the United Kingdom) according to the manufacturer's instructions.

### 2.12 Statistical Analysis

Results are presented as mean ± standard error of the mean (SEM) of each group. Statistical significance between mean values was assessed using the two‐way ANOVA followed by Tukey's multiple comparison or Mann–Whitney test and *t*‐tests, as appropriate. *p* values ≤ 0.05 were considered significant. All experiments were repeated at least 3 times. GraphPad Prism 7.0 was used for statistical analysis.

## 3 Results

### 3.1 PA Toxicity in rHep

**Figure 1:**
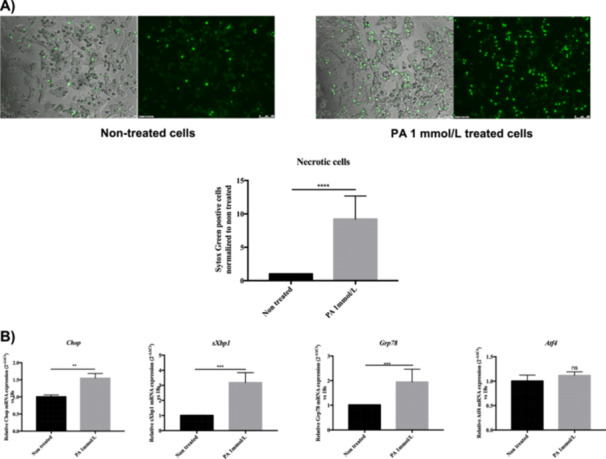
Palmitic acid cytotoxicity in primary rat hepatocytes. (A) Necrosis in primary rHep treated with PA (1 mmol/L) for 24 h determined by Sytox Green staining. Scale bar: 100 µm. (B) mRNA levels of ER stress markers *Chop, sXbp1, Grp78*, and *Atf4* were measured in rHep 8 h after treatment with PA (1 mmol/L) using 18S as a housekeeping gene. Data are shown as mean ± SEM (*n* ≥ 3). *p < 0.05, **p < 0.01, ***p < 0.001.

PA is a saturated long‐chain fatty acid that is toxic to hepatocytes [1]. PA at 1 mmol/L induces necrotic cell death in > 80% of hepatocytes. In nontreated hepatocytes, < 5% cell death was observed (Figure 1A). PA was not toxic at 0.25 mmol/L and showed minor toxicity at 0.5 mmol/L (data not shown). PA did not increase caspase‐3 activity, indicative of apoptotic cell death, at any of the concentrations tested (data not shown). PA also induced ER stress as demonstrated by the induction of several ER stress markers that initiate the unfolded protein response (UPR), such as Chop, sXbp1, and Grp78, without significantly inducing Atf4 expression (Figure 1B).

### 3.2 PA Increases the Generation of ROS and Induces Depolarization of the Mitochondrial Membrane in rHep

**Figure 2:**
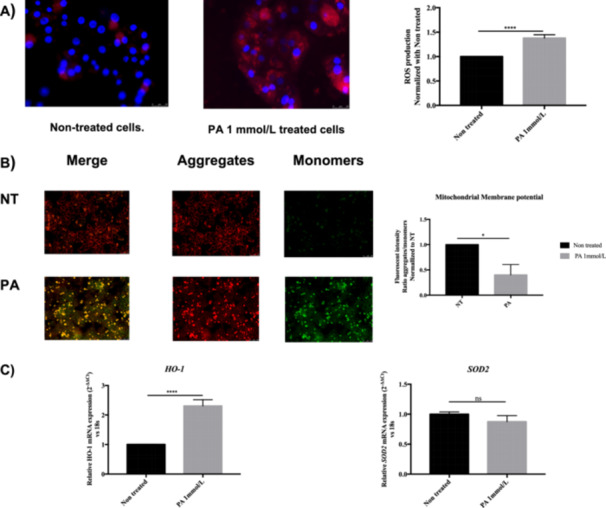
Oxidative stress induced by palmitic acid in primary rat hepatocytes. (A) Reactive oxygen species generation in mitochondria increased in PA‐treated cells (1 mmol/L) after 4 h of treatment. Scale bar: 20 µm. (B). Mitochondrial membrane depolarization induced by PA (1 mmol/L) after 12 h of treatment. Scale bar: 100 µm. (C) *HO‐1* and *SOD2* mRNA expression. 18S was used as a housekeeping gene. Data are shown as mean ± SEM (*n* ≥ 3). *p < 0.05, **p < 0.01, ***p < 0.001.

Previous reports have demonstrated that OxS plays an important role in the development of MASLD and NASH due to excessive lipid exposure. Therefore, the generation of ROS was analyzed in rHep after treatment with PA (1 mmol/L) using MitoSOX dye. As shown in Figure 2A, PA significantly increased ROS generation compared to nontreated cells. In addition, the polarization of the mitochondria was compromised by PA, as shown in Figure 2B, where PA decreased the aggregates (red)/monomers (green) ratio, indicating mitochondrial membrane depolarization. Furthermore, PA significantly increased the expression of the antioxidant enzyme heme oxygenase‐1 (Ho‐1) but had no effect on the expression of superoxide dismutase‐2 (Sod2; Figure 2C).

### 3.3 Evaluation of Toxicity and Selection of the Optimal Concentration of CV

**Figure 3:**
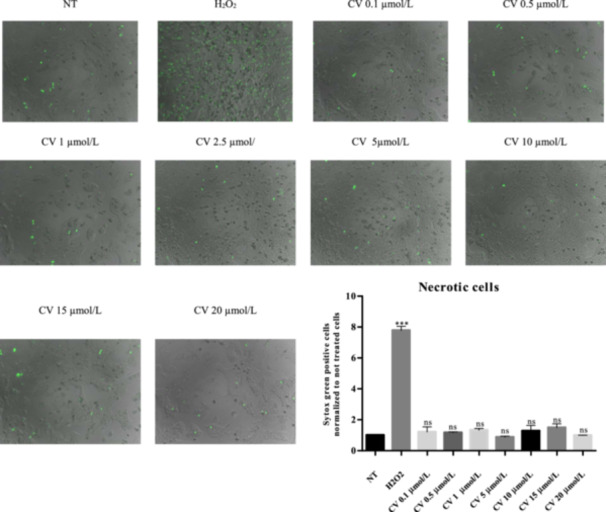
Determination of the optimal concentration of carvedilol. Sytox Green staining: carvedilol did not induce toxicity in rHep in the range of 0.1–20 μmol/L; scale bar: 100 μm. 10 µmol/L was the concentration selected for all subsequent experiments. *n* = 3–4. Data are shown as mean ± SEM (*n* ≥ 3). *p < 0.05, **p < 0.01, ***p < 0.001.

The toxicity of CV on rHep was determined using Sytox Green staining as an indicator of necrotic cell death and caspase‐3/7 activity assay as an indicator of apoptotic cell death to determine the optimal concentration of CV. CV was not toxic to primary hepatocytes at concentrations up to 20 µmol/L (< 5% dead cells as determined by Sytox Green staining and no increase of caspase‐3/7 activity). In preliminary experiments, the protective effect of different concentrations of CV was tested. A minor protective effect of CV was already noted at 2.5 µmol/L, and maximal protection was observed at 10 µmol/L (data not shown). Accordingly, we used a concentration of 10 µmol/L for all subsequent experiments (Figure 3).

### 3.4 CV Protects rHep Against Necrotic Cell Death Induced by PA

**Figure 4:**
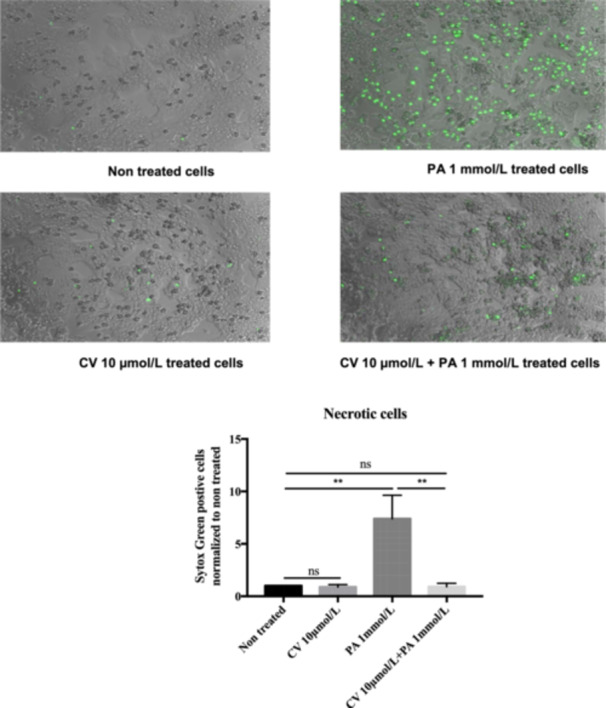
Necrotic cell death induced by palmitic acid (1 mmol/L) is reduced by carvedilol (10 µmol/L). Palmitic acid increased cell death sevenfold compared to the control, and this increase was abolished to control levels by carvedilol. Hepatocyte cell death was determined using Sytox Green staining. Scale bar: 100 μm. (*n* = 3–4).

CV abolished PA‐induced cell death in rHep at a concentration of 10 µmol/L (Figure 4). To validate the protective effect of CV on human liver cells, we also tested the effect of CV on palmitate‐induced apoptotic cell death in the human hepatoma cell line HepG2. PA‐induced caspase‐3/7 activity was inhibited by 10 µmol/L CV in HepG2 cells (data not shown).

### 3.5 CV Significantly Reduces the Generation of ROS and Restores Mitochondrial Membrane Potential

**Figure 5:**
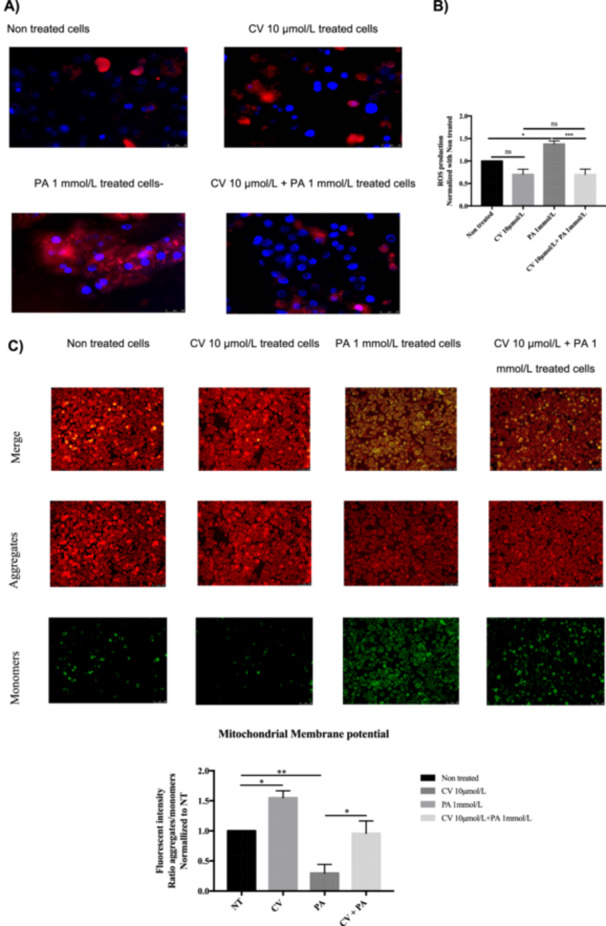
Carvedilol reduces palmitic acid‐induced ROS generation and improves mitochondrial membrane polarization in rHep. (A–B) CV reduces ROS generation in mitochondria in PA‐treated cells after 4 h of treatment by approx. 50% compared with PA‐treated cells (MitoSOX staining). (C) Mitochondrial membrane depolarization induced by PA (1 mmol/L) after 12 h of treatment in rHep was attenuated by CV. Red emission indicates mitochondrial membrane polarization (aggregates), and green emission indicates mitochondrial membrane depolarization (monomers). Statistical analysis was done by calculating the aggregates/monomer fluorescence ratio. Scale bar: 100 μm. Data are shown as mean ± SEM (*n* ≥ 3). *p < 0.05, **p < 0.01, ***p < 0.001.

CV has antioxidant properties, and PA treatment induces ROS generation. Therefore, the effect of CV on PA‐induced ROS generation was investigated. CV significantly reduced PA‐induced mitochondrial ROS production (Figure 5A,B). Additionally, since ROS can compromise mitochondrial integrity and function, mitochondrial depolarization was analyzed using JC‐10 dye. CV attenuated the PA‐induced mitochondrial depolarization, indicated by the increased JC‐10 aggregates (red)/monomers (green) ratio (Figures 2B and 5C), indicating that CV reduced PA‐induced ROS generation and restored mitochondrial membrane polarization.

### 3.6 CV Modulates the Expression of Antioxidant Enzymes in the Presence of PA

**Figure 6:**
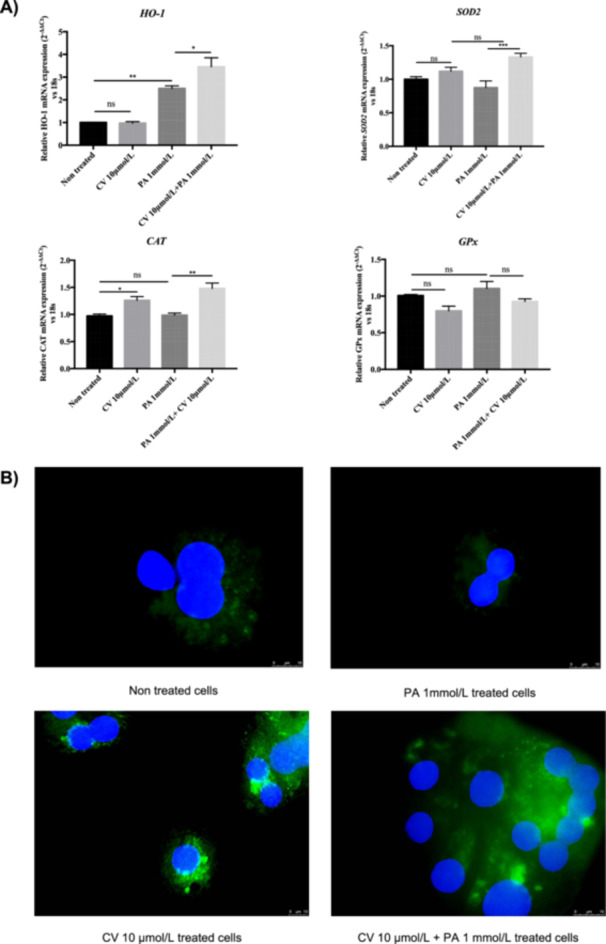
Carvedilol modulates the expression of antioxidant enzymes in rHep in the presence of palmitic acid. (A) CV (10 μmol/L) induced *Sod2* (+ 50%), *Cat* (+ 50%), and *Ho‐1* (+ 80%) mRNA expression but not *GPx* in the presence of PA after 8 h of treatment in rHep. mRNA levels were measured by real‐time quantitative PCR and normalized with 18S. (B) Immunofluorescence of *Sod2*. Magnification 40×, scale bars: 10 μm. Data are shown as mean ± SEM. *p < 0.05; **p < 0.01; ***p < 0.001; ns indicates *p* > 0.05. *n* = 3–4.

Since intracellular ROS levels were decreased by CV in the presence of PA as shown in Figure 4, we analyzed whether CV can modulate the expression of antioxidant enzymes. As shown in Figure 6, PA alone increased mRNA expression of the antioxidant enzyme HO‐1 compared to nontreated cells (NT). CV further increased PA‐induced expression of *Ho‐1* (Figure 6A). CV increased the expression of the mitochondrial antioxidant gene *SOD2* at the mRNA level in the presence of PA compared to PA alone, but CV did not affect *Sod2* expression in the absence of PA. CV increased the expression of the peroxisomal antioxidant enzyme catalase both in the absence and in the presence of PA. The expression of glutathione peroxidase (*GpPx*) was not changed by either CV or PA (Figure 6A). PA alone did not affect the expression of *Sod2*, catalase, or glutathione peroxidase compared to nontreated control rHep. To confirm the effect of CV on *Sod2* mRNA expression at the protein level, we performed immunofluorescence. CV increased the protein level of Sod2 both in control cells and in PA‐treated cells (Figure 6B).

### 3.7 The Protective Effect of CV Is Not Related to the Reversal of ER Stress

**Figure 7:**
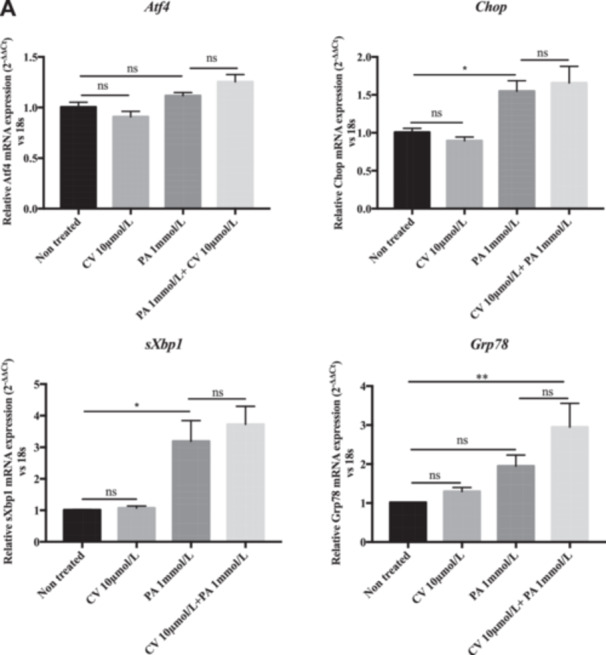
ER stress markers are not modulated by carvedilol treatment in palmitic acid‐treated primary rHep. (A) Primary rHep were exposed to PA (1 mmol/L) and CV (10 μmol/L) for 8 h. mRNA levels of Atf4, Chop, sXbp1, and Grp78 were evaluated by real‐time quantitative PCR. 18S was used as a housekeeping gene. Data are shown as mean ± SEM. *p < 0.05; **p < 0.01; ***p < 0.001; ns indicates *p* > 0.05. *n* = 3–4.

PA induces ROS generation, and since ER stress is linked to excessive ROS generation [1], we investigated the effect of PA and CV on ER stress‐related markers of the UPR (Figure 1). PA induced the expression of the ER stress markers Chop and sXbp1 (Figure 7). CV did not reverse the PA‐induced increase in the mRNA expression of the ER stress markers (*Chop* and *sXbp1*). The expression of the Grp78 gene, which encodes for the chaperone BiP, appeared to be increased in the PA + CV group compared to the PA group; however, this difference did not reach statistical significance (Figure 7), suggesting that the protective effect of CV is not related to reduction of PA‐induced ER stress.

### 3.8 CV Does Not Affect Lipid Accumulation in rHep

**Figure 8:**
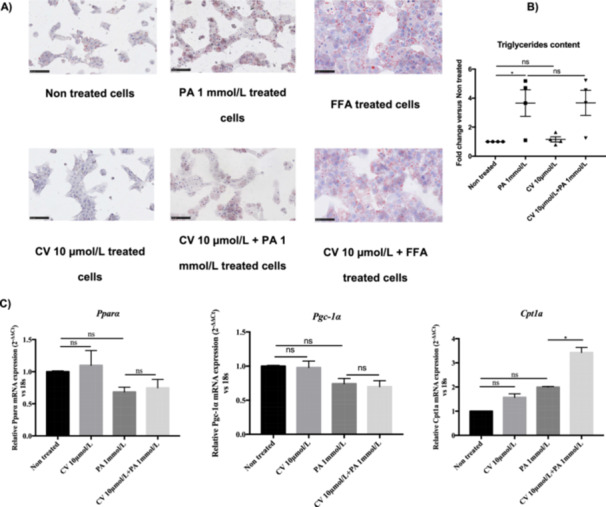
Carvedilol does not modulate intracellular lipid content. (A) Cells were pretreated with carvedilol (10 µmol/L) for 30 min and subsequently exposed to palmitic acid (1.0 mmol/L) or the combination of oleate and palmitate for 12 h. Lipid droplets were detected by Oil Red O staining. Magnification 20×, scale bars: 100 μm. (B) Intracellular triglyceride content was quantified in rHep. (C) mRNA expression of lipid metabolism‐related genes (Pparα, Pgc‐1α, and Cpt1a) was determined by real‐time quantitative PCR after 8 h of treatment with CV and/or PA. 18S was used as a housekeeping gene. Data are shown as mean ± SEM. *p < 0.05; **p < 0.01; ***p < 0.001; ns indicates *p* > 0.05. *n* = 3–4.

β‐AR signaling is involved in the catabolism of lipids via fatty acid β‐oxidation. Therefore, we next investigated whether CV affects lipid accumulation. As shown in Figure 8A, PA leads to minor lipid accumulation compared to the combination of the FFAs oleate and palmitate. The combination of oleate and palmitate induces extensive lipid droplet accumulation and is used as a positive control for lipid accumulation. CV did not change the lipid accumulation in PA‐treated rHep. PA slightly increased total TG content in rHep, and CV did not change this (Figure 8B). The expression levels of the lipid metabolism‐related genes PPARα, Pgc‐1, and Cpt1a were also examined. PA tended to decrease the expression of PPARα and Pgc‐1 in rHep, but this decrease was not statistically significant. Moreover, CV did not change the expression of these genes both in the absence and in the presence of PA (Figure 8C). Likewise, PA tended to increase the expression of Cpt1a, but this increase was not statistically significant. However, the combination of CV and PA strongly increased (threefold) the expression of Cpt1a compared to nontreated rHep (Figure 8C).

## 4 Discussion

Exposure of hepatocytes to high levels of (toxic) lipids is a risk factor in the development of liver diseases such as MASLD and NASH. PA is the most common saturated FFA in the human body and accounts for approximately 20%–30% of total FFAs [2]. It is a lipotoxic fatty acid and induces detrimental effects in the parenchymal cells of the liver, for example, hepatocytes [28, 30].

In the present study, we demonstrate that CV, an antagonist of β‐ and α‐ARs used to treat hypertension and congestive heart failure [31], abolishes cell necrosis caused by PA in rHep. Previously, we showed that PA induces ROS generation in primary hepatocytes and HepG2 cells [28], as well as ER stress and cell necrosis [1]. In the present study, we show that CV reduces PA‐induced OxS and necrosis and restores mitochondrial membrane polarization in primary hepatocytes.

CV, besides being an antagonist of ARs, also has intrinsic antioxidant properties. CV can directly neutralize ROS such as superoxide anions since CV contains a carbazole ring, a tricyclic structure that is crucial for its antioxidant activity. This moiety can donate electrons or hydrogen atoms to free radicals and effectively neutralize the free radicals [18]. In the pathogenesis of atherosclerosis, CV showed ROS‐scavenging activity, which was linked to the carbazole moiety of CV [18, 19]. CV also decreases OxS in human myocardial infarction (Nakamura et al. 2002). Although CV can be a direct ROS scavenger, we also investigated the effect of CV on antioxidant enzyme gene expression since these enzymes are considered important defense systems against OxS (Rodriguez et al. 2004). Carvedilol increases the expression of *HO‐1* and *SOD2* only in the presence of palmitate and not, or hardly (*CAT*), in the absence of palmitate. Palmitate alone only induces the expression of *HO‐1*. Therefore, we can conclude that CV increases antioxidant gene expression in the presence of palmitate, that is, only in the presence of palmitate‐induced oxidant stress. This makes sense since an antioxidant response is only useful in the presence of oxidant stress. We did not investigate the transcriptional mechanisms responsible for the synergistic induction of antioxidant gene expression by CV in the presence of palmitate. Effects of CV on antioxidant enzyme expression in hepatocytes have been reported before [32] and confirm our observations. Also, in other cell populations, CV has antioxidant properties; for example, in retinal pigment epithelial cells, CV activates the Nrf2/ARE signaling pathway [33]. Moreover, CV tended to increase the expression of *HO‐1* at both protein and mRNA levels in bovine aortic endothelial cells under TNF‐α stimuli [34] and in the human retinal pigment epithelial cell line ARPE‐19 under high‐glucose treatment [33]. Additionally, in an in vivo model of streptozotocin‐induced hyperglycemia and diabetes type I in rats, CV showed beneficial effects on diabetic cardiomyopathy and increased protein expression of *SOD2, SOD1*, and catalase in heart and skeletal muscle [35].

ROS interact with biological membranes, leading to modifications that compromise their function and, as a consequence, can lead to organellar dysfunction, for example, mitochondrial or ER dysfunction [36, 37]. The mitochondria are both source and target of ROS (Murphy et al. 2016). ROS can cause mitochondrial depolarization, leading to increased cytoplasmic Ca^2+^ concentration and, ultimately, cell death [38]. In our previous studies, we showed that PA induces high levels of mitochondrial ROS and mitochondrial depolarization [28]. In our present study, we show that CV restored mitochondrial membrane potential. Previously, we showed that palmitate (1 mmol/L) severely affects mitochondrial respiration and increases ROS generation (Geng Y et al. 2020). In the present study, we show that palmitate‐induced membrane depolarization and mitochondrial ROS production are attenuated by CV, suggesting that mitochondrial dysfunction and ROS generation are the cause of necrosis and that CV protects against mitochondrial dysfunction and consequent cell death.

Saturated fatty acids such as palmitate can change the membrane composition of the ER and/or disrupt the protein folding machinery, leading to ER stress and the activation of the UPR [39, 40, 41]. In normal conditions, the UPR is a cytoprotective response; however, in conditions of (prolonged) stress, the UPR can activate cell death pathways, leading to apoptotic and/or necrotic cell death [42]. In human HepG2 hepatoma cells, PA can induce the expression of the chaperone protein GRP78 and the UPR mediator CHOP as well as apoptosis [28, 40]. In rHep, PA induces ER stress via activation of different effector arms of the UPR: inositol‐requiring enzyme 1α (IRE1α) and protein kinase RNA (PKR)‐like kinase (PERK), leading to necrotic cell death [1]. We confirmed these observations in our study; however, CV did not reduce the mRNA expression of *Chop* or *sXbp1*—downstream regulators of the UPR effector arms mentioned above. These results indicate that CV does not directly reduce ER stress and that the protective effect of CV against necrotic cell death in primary hepatocytes is due to other mechanisms, for example, prevention of ROS‐induced damage. In this respect, it is important to note that the antioxidant capacity of CV is higher than that of the well‐known antioxidant vitamin E (30–80 times more potent) [43].

It is known that β‐AR stimuli regulate lipolysis in hepatocytes via the activation of the cAMP/PKA pathway [22, 44, 45, 46]. Considering that CV is an antagonist of β‐ARs, we analyzed the effect of CV on oleate + palmitate‐induced lipid accumulation in hepatocytes. We did not observe an effect of CV on PA‐induced lipid accumulation. Interestingly, we observed that CV in the presence of PA increased the expression of Cpt1a. Cpt1 is the gene that encodes the enzyme carnitine palmitoyl transferase, which is involved in the catabolism of lipids. It is located in the outer mitochondrial membrane and is involved in the conversion of acyl‐CoA esters into carnitine esters to facilitate their entrance into the matrix of the mitochondria, thus fueling β‐oxidation [47]. Thus, CV may also contribute to reducing the levels of lipotoxic FFAs such as palmitate by increasing the expression of Cpt1 and facilitating the removal of PA via β‐oxidation in mitochondria. A limitation of our study is that we use an in vitro model of PA toxicity. Nevertheless, in this model, we observed cell death and lipid accumulation in response to fatty acids, two hallmarks of MASLD. Moreover, this in vitro model is widely used in studies of lipotoxicity. Primary hepatocytes are more representative than hepatoma cell lines because they are more differentiated in the early stage of culture. Therefore, all our experiments lasted for a maximum of 24 h in which the differentiated state of the hepatocytes was fully maintained. Nevertheless, we also tested CV on the human hepatoma cell line HepG2 and observed similar protective effects as on rHep, although, in HepG2 cells, PA causes apoptotic cell death, not necrotic cell death. These results are in line with a recent study by Moterlini et al., investigating the toxicity of fatty acids in hepatoma cells. In this study, toxicity correlated with lack of lipid droplet formation, whereas protection against lipotoxicity correlated with abundant lipid droplet formation [48]. Another limitation of our study is that we used only one concentration of CV. We selected 10 µmol/L because this concentration is widely used for in vitro studies and did not induce any toxicity. Furthermore, in preliminary studies, we determined that maximal protection was obtained using 10 µmol/L. It is difficult to compare concentrations between in vitro and in vivo models. Iwaki et al. investigated the plasma concentrations after intravenous administration of CV to rats and observed plasma concentrations that were in the range of the concentrations we used in vitro [49]. Therefore, we believe that the concentrations of CV we used in vitro are comparable to the concentrations that can be reached in vivo. Although CV appears to be an attractive candidate to treat patients with MASLD, or any other OxS‐related disorder, additional research is necessary to elucidate the optimal dosage and administration of CV, its uptake and metabolism in the liver, pharmacokinetics and pharmacodynamics, and any side effects. Previous studies have investigated the use of the antioxidant vitamin E in the treatment of MASLD with some beneficial results [50].

## 5 Conclusion

This study revealed that CV protected against palmitate‐induced cell death in hepatocytes via scavenging of ROS without modulating lipid accumulation or reducing ER stress. To establish whether CV can be used as a treatment for diseases related to OxS such as MASLD and NASH, additional studies of toxicity, pharmacokinetics, and pharmacodynamics in human trials are necessary.

## Author Contributions

**Sandra Serna Salas:** conceptualization, formal analysis, investigation, methodology, writing–original draft. **Turtushikh Damba:** conceptualization, formal analysis, investigation, methodology, writing–original draft. **Manon Buist‐Homan:** formal analysis, investigation, methodology, validation. **Han Moshage:** conceptualization, formal analysis, project administration, supervision, validation, manuscript review and editing.

## Conflicts of Interest

The authors declare no conflicts of interest.

## Data Availability

The data that support the findings of this study are available from the corresponding author upon reasonable request.
